# Prevalence of diabetic cardiomyopathy in patients with type 2 diabetes in a large academic medical center

**DOI:** 10.1186/s12916-024-03401-3

**Published:** 2024-05-14

**Authors:** Iwona Swiatkiewicz, Neeja T. Patel, MaryAnn Villarreal-Gonzalez, Pam R. Taub

**Affiliations:** Division of Cardiovascular Medicine, University of California San Diego, La Jolla, CA 92037 USA

**Keywords:** Diabetic cardiomyopathy, Heart failure, Echocardiography, Diabetes, Comorbidities, Outcome

## Abstract

**Background:**

Diabetic cardiomyopathy (DbCM) is characterized by asymptomatic stage B heart failure (SBHF) caused by diabetes-related metabolic alterations. DbCM is associated with an increased risk of progression to overt heart failure (HF). The prevalence of DbCM in patients with type 2 diabetes (T2D) is not well established. This study aims to determine prevalence of DbCM in adult T2D patients in real-world clinical practice.

**Methods:**

Retrospective multi-step review of electronic medical records of patients with the diagnosis of T2D who had echocardiogram at UC San Diego Medical Center (UCSD) within 2010–2019 was conducted to identify T2D patients with SBHF. We defined “pure” DbCM when SBHF is associated solely with T2D and “mixed” SBHF when other medical conditions can contribute to SBHF. “Pure” DbCM was diagnosed in T2D patients with echocardiographic demonstration of SBHF defined as left atrial (LA) enlargement (LAE), as evidenced by LA volume index ≥ 34 mL/m^2^, in the presence of left ventricular ejection fraction (LVEF) ≥ 45%, while excluding overt HF and comorbidities that can contribute to SBHF.

**Results:**

Of 778,314 UCSD patients in 2010–2019, 45,600 (5.9%) had T2D diagnosis. In this group, 15,182 T2D patients (33.3%) had echocardiogram and, among them, 13,680 (90.1%) had LVEF ≥ 45%. Out of 13,680 patients, 4,790 patients had LAE. Of them, 1,070 patients were excluded due to incomplete data and/or a lack of confirmed T2D according to the American Diabetes Association recommendations. Thus, 3,720 T2D patients with LVEF ≥ 45% and LAE were identified, regardless of HF symptoms. In this group, 1,604 patients (43.1%) had overt HF and were excluded. Thus, 2,116 T2D patients (56.9% of T2D patients with LVEF ≥ 45% and LAE) with asymptomatic SBHF were identified. Out of them, 1,773 patients (83.8%) were diagnosed with “mixed” SBHF due to comorbidities such as hypertension (58%), coronary artery disease (36%), and valvular heart disease (17%). Finally, 343 patients met the diagnostic criteria of “pure” DbCM, which represents 16.2% of T2D patients with SBHF, i.e., at least 2.9% of the entire T2D population in this study.

**Conclusions:**

Our findings provide insights into prevalence of DbCM in real-world clinical practice and indicate that DbCM affects a significant portion of T2D patients.

## Background

Diabetic cardiomyopathy (DbCM) is a primary myocardial disease with altered myocardial structure and fibrosis which results from diabetes-associated metabolic alterations [1–5]. DbCM is an asymptomatic stage B of heart failure (SBHF) in patients with diabetes which is characterized by a long subclinical phase of left ventricular (LV) remodeling before development of myocardial stiffness with LV dysfunction (LVD), most often LV diastolic dysfunction (LVDD) [3, 6, 7]. DbCM is associated with a high risk of progression to overt heart failure (HF), i.e., stage C of HF (SCHF) or stage D of HF (SDHF), usually with preserved LV ejection fraction (LVEF), and adverse outcome with increased morbidity and mortality [8–11].

DbCM is diagnosed by echocardiographic demonstration of abnormal cardiac structure and/or performance in patients with diabetes in the absence of substantial comorbidities other than diabetes which can contribute to SBHF such as essential systemic hypertension (HTN), especially poorly controlled, coronary artery disease (CAD), congenital heart disease, valvular heart disease (VHD), and other primary and secondary cardiomyopathies [12, 13]. DbCM may start with SBHF characterized by the presence of structural cardiac abnormality (e.g., LV hypertrophy, chamber enlargement), evidence of increased filling pressure, and/or elevated biomarkers (natriuretic peptide or persistently elevated troponin levels) [14, 15]. Specific criteria for defining DbCM, however, have not been well established. In clinical practice, a diagnosis of DbCM can be evidenced by presence of various echocardiographic abnormalities including LV hypertrophy (LVH) and/or enlargement, LV diastolic and/or systolic dysfunction, and left atrial (LA) enlargement (LAE) [8–10, 12, 13, 15–17]. In patients with preserved LVEF, key structural alterations include an increase in LA size and volume, and/or an increase in LV mass [15]. LAE represents a widely used and documented echocardiographic marker of both the severity and chronicity of LVDD with increased LV filling pressure and magnitude of LA pressure elevation, common to many clinical studies protocols and centralized databases [11, 13, 15, 18–27]. LAE was also shown to have prognostic value for HF development in patients with type 2 diabetes (T2D) and SBHF [16, 24, 27].

DbCM is a common sequela of diabetes, which can affect up to 17% of individuals with diabetes [8, 9, 12, 13, 16]. The prevalence of DbCM in patients with diabetes in a real-world clinical practice is not well established, mainly due to non-homogeneity of studied populations and a lack of consensus on criteria for defining DbCM. Current data from small studies suggests a prevalence of DbCM of 1.1% in the general population and up to 17% of patients with diabetes with morbidity and mortality approaching 31% over 10 years [8, 9]. Previous studies on the prevalence of DbCM in T2D patients have been limited by heterogenous populations, small sample size, and heterogeneity of clinical and echocardiographic criteria for the diagnosis of DbCM [8–10, 16]. Existing results indicate an association between T2D and a risk of developing HF [28–32]. Specifically, a twofold higher incidence of HF in male and fivefold higher incidence in female patients with T2D compared to subjects without T2D, especially in older patients, were reported [28]. The risk of developing symptomatic HF in T2D patients over a long-time observation may be even higher in younger individuals (e.g., 11-fold in individuals < 45 years old [29]). However, data on a progression of asymptomatic DbCM to symptomatic HF in T2D patients remain limited [8, 10, 16]. Thus, there is a clear unmet need to collect further data on the prevalence of DbCM using a rigorous methodology, which may be useful for developing optimal approaches to manage patients with T2D.

This study aims to evaluate a prevalence of DbCM in patients with T2D in a real-world clinical practice. Specifically, we sought to determine the prevalence of asymptomatic SBHF related solely to T2D among T2D patients who have not yet progressed to overt HF.

## Methods

### Study design and database development

We conducted a longitudinal cohort study (*Diabetic CardioMyopathy-Heart Failure Study*, i.e., DbCM-HF Study) including patients undergoing medical care at the University of California San Diego Medical Center, La Jolla, CA, USA (UCSD) who were diagnosed with T2D and had transthoracic echocardiogram (TTE) in the period from 1 January 2010 to 31 December 2019. For brevity, when we refer to patients with T2D we consider patients who had T2D and underwent TTE. The prevalence of DbCM has been determined based on retrospective review of patients’ Epic electronic medical records (EMRs). To identify T2D patients with SBHF we applied a multi-step approach including the following components: an identification of patients with the diagnosis of T2D who had TTE, selection of T2D patients with echocardiographic features of LVD suggesting DbCM, and exclusion of T2D patients with LVD who had overt symptomatic HF (i.e., SCHF or SDHF). Finally, from the group of T2D patients with SBHF, we excluded patients with SBHF and medical conditions other than T2D that can contribute to SBHF. With this approach, we identified the population of T2D patients who meet the diagnostic criteria of “pure” DbCM that was defined as an asymptomatic SBHF associated solely with T2D.

To achieve the high level of generalizability of study results, we developed a study protocol that is characterized by rigorous and well-defined inclusion and exclusion criteria, large sample size, availability of various clinical and echocardiographic data for identifying and characterizing patients with DbCM through an access to comprehensive EMRs, and inclusion of demographically diverse population of patients undergoing high-quality medical care in a real-world clinical practice according to the current evidence-based guidelines.

For the purpose of our analysis, we defined “pure” DbCM as SBHF in T2D patient that is associated solely with T2D. Moreover, we defined “mixed” SBHF in T2D patient when comorbidities other than T2D can contribute to SBHF. Specifically, “pure” DbCM was diagnosed in T2D patients based on echocardiographic demonstration of SBHF defined as LAE in the presence of LVEF ≥ 45%, while excluding overt HF and comorbidities that can contribute to SBHF [12–15, 18]. LAE was defined as LA volume index (LAVI) ≥ 34 mL/m^2^ [15, 19, 20, 33, 34]. LVEF ≥ 45% was classified in our analysis as preserved LVEF [14, 19, 20]. “Mixed” SBHF was diagnosed in patients with the diagnosis of T2D and other comorbidities based on echocardiographic demonstration of SBHF defined as LAE in the presence of LVEF ≥ 45%, while excluding overt HF. Consequently, the population of patients with “mixed” SBHF includes T2D patients with echocardiographic features of SBHF suggesting DbCM and comorbidities that can contribute to SBHF. For clarity, we used the term SBHF when T2D patient without overt HF had LAE in the presence of LVEF ≥ 45% in echocardiography. We also used the term LVD when T2D patient had LAE in the presence of LVEF ≥ 45% in echocardiography, regardless of the presence of HF symptoms.

**Fig. 1:**
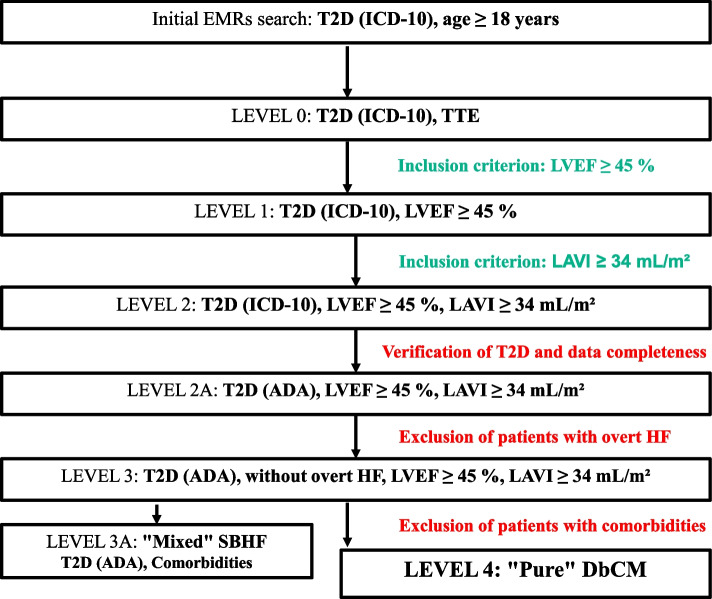
Design of study and development of study database. Abbreviations: ADA – American Diabetes Association; DbCM – diabetic cardiomyopathy; EMRs – Epic electronic medical records; HF – heart failure; ICD-10 – International Classification of Diseases, Tenth Revision; LAVI – left atrial volume index; LVEF – left ventricular ejection fraction; SBHF – stage B heart failure; T2D – type 2 diabetes; TTE – transthoracic echocardiogram

Patients satisfying eligibility criteria for the DbCM-HF study were identified based on multi-step search of EMRs (Fig. 1). Specifically, we selected eligible patients by applying step-by-step multiple search criteria to generate multiple levels of database. Patients were initially eligible for the study if they had (Table 1): (1) Diagnosis of T2D according to the International Classification of Diseases, Tenth Revision (ICD-10) and (2) Age ≥ 18 years (Fig. 1, Initial search), (3) Transthoracic echocardiogram (TTE) performed and echocardiogram report available (Fig. 1, Level 0). In the next step, we applied echocardiographic diagnostic criteria for DbCM and identified the population of patients with the diagnosis of T2D and echocardiographic demonstration of LVD suggesting DbCM, i.e., preserved LV systolic function defined as LVEF ≥ 45% (Fig. 1, Level 1) and LAE defined as LAVI ≥ 34 mL/m^2^ (Fig. 1, Level 2).

**Table 1:** Inclusion and exclusion criteria

**Inclusion criteria**
• Diagnosis of type 2 diabetes
• Age ≥ 18 years
• Echocardiogram performed and echocardiogram report available
• Left ventricular ejection fraction ≥ 45%
• Left atrial volume index ≥ 34 mL/m^2^
**Exclusion criteria**
• Prior diagnosis of overt symptomatic heart failure (stage C or stage D heart failure)
• Any prior echocardiographic measurement of left ventricular ejection fraction < 40%
• Coronary artery disease (≥ 50% stenosis of any major coronary artery)
• History of acute coronary syndrome
• Previous or planned coronary revascularization
• Moderate, severe, and uncontrolled mild hypertension, i.e., systolic blood pressure ≥ 140 and/or diastolic blood pressure ≥ 90 mmHg despite antihypertensive treatment
• History of hospitalization for hypertensive emergency
• History of stroke related to hypertension and/or atherosclerotic vascular disease
• History of severe valvular disease or moderate valvular disease requiring intervention
• History of congenital, infective, toxic (chemotherapy, radiation, methamphetamine, or alcohol), infiltrative (amyloidosis, sarcoidosis, or hemochromatosis), post-partum, or hypertrophic cardiomyopathy
• History of myocarditis induced by active autoimmune disease
• Pulmonary arterial hypertension (WHO Group 1)
• Severe chronic lung and pulmonary vascular diseases
• Severe cardiac involvement in patients with liver cirrhosis
• Severe disease with short life expectancy (e.g., active disseminated neoplastic disease)
• History of substance abuse
• Incomplete clinical and/or echocardiographic data

Then, we reviewed EMRs of each individual patient included at Level 2 to verify data completeness and validate a diagnosis of T2D following the American Diabetes Association (ADA) recommendations [35, 36] (Fig. 1, Level 2). With this approach, we identified a group of patients with confirmed diagnosis of T2D and complete data, who had echocardiographic features of LVD suggesting DbCM (Fig. 1, Level 2A). Then, we excluded patients with overt symptomatic HF (i.e., patients with SCHF or SDHF) to select asymptomatic patients with SBHF suggesting DbCM (Table 1; Fig. 1, Level 3). Out of them, in the final step of our approach, T2D patients with “mixed” SBHF, i.e., T2D patients with comorbidities that can contribute to SBHF, were identified and excluded (Table 1; Fig. 1, Level 3A). The medical conditions that can contribute to SBHF included the comorbidities such as CAD, history of acute coronary syndrome or coronary revascularization, severe and moderate HTN, uncontrolled mild HTN, significant VHD, congenital heart disease, primary and secondary cardiomyopathies, prior measurement of LVEF < 40%, history of myocarditis, stroke related to HTN and/or atherosclerotic vascular disease, substance abuse, severe cardiac involvement in other medical conditions such as liver cirrhosis or lung and pulmonary vascular diseases, and others (Table 1) [37, 38]. With this approach, we selected T2D patients with SBHF who did not have medical conditions that can contribute to SBHF. Thus, we identified the final population of asymptomatic T2D patients with “pure” DbCM defined as SBHF associated solely with T2D (Fig. 1, Level 4).

Initial steps of EMRs search (from an initial search through Level 2 of database, Fig. 1) involved an automated bioinformatics search of the UCSD EMRs by applying multiple search criteria to identify initial population of patients satisfying eligibility criteria for the study. For this purpose, the following search criteria were used: (1) diagnosis of T2D (encoded as T2D according to ICD-10) in the period from 1 January 2010 through 31 December 2019; (2) age ≥ 18 years; (3) TTE performed from 1 January 2010 through 31 December 2019; (4) LVEF ≥ 45%; and (5) LAVI ≥ 34 mL/m^2^. The preliminary list of eligible patients (whose personal data were de-identified) was ordered according to the date of first (i.e., the earliest) TTE satisfying the eligibility criteria during the 10-year study period. The repetitive entries of the same patient during this period were excluded. In the next steps (from Level 2 to 4, Fig. 1), the EMRs of each patient were manually reviewed to identify eligible patients. All data included in the EMRs were available for review. Data extraction and data entries to pre-specified data-extraction forms, as well as initial identification of non-eligible patients were made by two trained young investigators (N.T.P. and M.V.G.) who have been working under a supervision of senior investigator with an expertise in internal medicine, cardiology, and clinical trials management (I.S.). Extracted data included the data on patient characteristics such as demographics, comorbidities, echocardiographic parameters measurements, lab tests and other diagnostic tests results, data related to T2D diagnosis, treatment and complications, and data related to the presence of overt HF. Data quality including accuracy, completeness, consistency, and relevance was assessed by senior investigator (I.S.) by reviewing the EMRs of each patient included at Level 2, 2A, 3, and 4 (Fig. 1). In this step non-eligible patients with incomplete data, unconfirmed T2D diagnosis, overt HF, and comorbidities that may contribute to SBHF were excluded, and patients with “pure” DbCM were ultimately identified and included (Level 4, Fig. 1). Any discrepancies were resolved by consensus through discussion involving two senior investigators (I.S. and P.R.T.) and two young investigators (N.T.P. and M.V.G.).

Data on the diagnosis of overt symptomatic HF were acquired through the EMRs review. Overt symptomatic HF was defined as: 1) the presence of HF symptoms corresponding to ≥ II NYHA class, i.e., shortness of breath at rest or during exertion and/or fatigue and signs of fluid retention such as pulmonary congestion or ankle swelling; data on HF symptoms were acquired at medical visits and recorded in the EMRs; 2) hospitalization for HF requiring documented clinical and/or radiologic evidence of clinical HF and congestion; specifically, HF hospitalization was defined as admission to hospital due to new or increasing symptoms and signs of HF including fluid retention or other objective evidence of HF, such as increasing dyspnea by one or more NYHA class(es), peripheral edema, bilateral post-tussive rales in at least lower third of lung fields, or ventricular gallop rhythm, in combination with a change in treatment to improve HF including parenteral use of diuretic [39]; data on HF hospitalization were acquired based on the record of hospitalizations in the EMRs, specifically the hospitalizations encoded as HF hospitalization.

Data on the comorbidities and past medical history were acquired through the EMRs review. CAD was diagnosed based on the presence of ≥ 50% stenosis of any major coronary artery. Diagnosis of HTN was made based on systolic blood pressure (BP) ≥ 140 mmHg and/or diastolic BP ≥ 90 mmHg, or use of antihypertensive medication(s) [40]. Moderate, severe, and mild HTN were classified according to the recommendations [40]. Patients with moderate HTN (defined as systolic BP values of 160–179 mmHg and/or diastolic BP values of 100–109 mmHg), severe HTN (defined as systolic BP ≥ 180 mmHg and/or diastolic BP ≥ 110 mmHg), and uncontrolled mild HTN despite antihypertensive treatment (defined as systolic BP of 140–159 mmHg and/or diastolic BP of 90–99 mmHg when BP measurements remain ≥ 140/90 mmHg after the diagnosis of HTN as noted in EMRs), were excluded. BP and resting heart rate measurements were done at medical visits under resting (after a 5-min rest) and an output was an average of three readings 1–2 min apart. History of stroke related to HTN and/or atherosclerotic vascular disease was an exclusion criterion. Cardiomyopathy was defined as a myocardial disorder in which the heart muscle is structurally and functionally abnormal in the absence of CAD, HTN, VHD, and congenital heart disease sufficient to explain the observed myocardial abnormality [41]. Cardiac involvement (cirrhotic cardiomyopathy), portopulmonary hypertension, and hepatopulmonary syndrome in patients with liver cirrhosis were individually assessed to determine patient eligibility. Cardiac involvement and pulmonary hypertension in patients with chronic lung and pulmonary vascular diseases (such as chronic obstructive pulmonary disease, interstitial pulmonary fibrosis, or chronic thromboembolic pulmonary hypertension) were individually assessed to determine patient eligibility. Patients with severe disease with short life expectancy (such as active neoplastic disease, etc.) were individually assessed to determine patient eligibility. Patients with incomplete clinical and/or echocardiographic data were excluded.

Patients with nonobstructive CAD, i.e., with < 50% stenosis of any major coronary artery were eligible. Patients with the diagnosis of mild HTN that was well controlled (i.e., when the measurements of systolic BP were < 140 mmHg and diastolic BP < 90 mmHg at medical visits after the diagnosis of HTN as noted in the EMRs in a long-term observation) were eligible. Patients with a history of cryptogenic or cardioembolic stroke were eligible. Patients with chronic kidney disease (CKD) were eligible. CKD was defined as glomerular filtration rate < 60 mL/min/1.73 m^2^ and < 50 mL/min/1.73 m^2^ (as calculated by the MDRD formula) for persons aged 45–64 and 65–80, respectively, which have existed for 3 months or longer [42].

With our approach, we generated a project-specific database of T2D patients with “pure” DbCM which includes demographic, clinical, biochemical, and echocardiographic variables. Based on analyzing the DbCM database, we developed clinical and echocardiographic characteristics of the population of T2D patients with “pure” DbCM.

The study was conducted in accordance with the Declaration of Helsinki. Approval from the ethics committee of UCSD Human Research Protections Program was obtained (IRB 200205/2020). Written informed consent for the participation in the study was waived given retrospective nature of this analysis.

### Echocardiography

Echocardiographic data were acquired from echocardiographic reports and TTE recordings that were included in the EMRs of patients undergoing medical care at the UCSD in the 10-year period, i.e., from 1 January 2010 to 31 December 2019. Echocardiographic reports and recordings were analyzed by two cardiologists (I.S., P.R.T.) blinded to the clinical outcomes and biomarker levels to describe echocardiographic characteristics of patients with “pure” DbCM.

Comprehensive two-dimensional and Doppler TTE were performed using commercially available ultrasound instruments. All echocardiographic examinations were performed as part of standard medical care. TTE was performed following the American Society of Echocardiography (ASE) recommendations [19, 20]. Measurements from three consecutive cardiac cycles were averaged.

Measurements of LV end-diastolic and LV end-systolic diameters were made using TTE parasternal long axis view with M-mode cursor positioned just beyond the mitral leaflet tips, perpendicular to the LV long axis. LV mass (LVM) and LVM index (LVMI) were calculated according to Deveraux formula. The values of LVMI above reference upper limits, i.e., 95 g/m^2^ in women and 115 g/m^2^ in men, were considered as indicating LVH [20].

LV volumes and LVEF were calculated using the biplane method of disks (modified Simpson’s rule) in two- and four-chamber views, which is the recommended method of choice for LVEF assessment in echocardiography [19, 20]. The principle underlying this method is that the total LV volume is calculated from the summation of a stack of elliptical disks. The height of each disk is calculated as a fraction of the LV long axis based on the longer of the two lengths from the two- and four-chamber views. The cross-sectional area of the disk is based on the two diameters obtained from the two- and four-chamber views.

Left atrial (LA) volume was measured from standard apical 4-chamber views at end-systole just before mitral valve opening. LA borders were traced using planimetry. The borders consisted of the walls of the LA excluding pulmonary veins and LA appendage. The biplane method of disks was used to calculate LA volume. LAVI was calculated by dividing LA volume by body surface area of subjects.

LV diastolic function was evaluated using the mitral valve inflow which was recorded by pulse-wave Doppler from TTE apical 4-chamber view. Peak E-wave (early transmitral flow) velocity and its deceleration time, and peak A-wave (transmitral flow during atrial systole) velocity were measured. In a portion of patients, data on tricuspid regurgitation velocity (TR) and E/E’ ratio, where E’ is diastolic mitral annular velocity by Tissue Doppler Imaging, were available. TR was measured by continuous-wave Doppler from TTE apical 4-chamber view. LV diastolic dysfunction (LVDD) was diagnosed according to ASE recommendations [19, 20, 33, 34].

### Statistical analysis

Statistical analyses were carried out using the Statistica 13.1 software (TIBCO Software Inc, California, USA). The Shapiro–Wilk test demonstrated non-normal distribution of the investigated data. Continuous variables were presented as medians with interquartile ranges. Categorical variables were expressed as the numbers and the percentages. Statistical significance was assumed at the level of *p* < 0.05.

## Results

### Patient flow and prevalence of diabetic cardiomyopathy

**Fig. 2:**
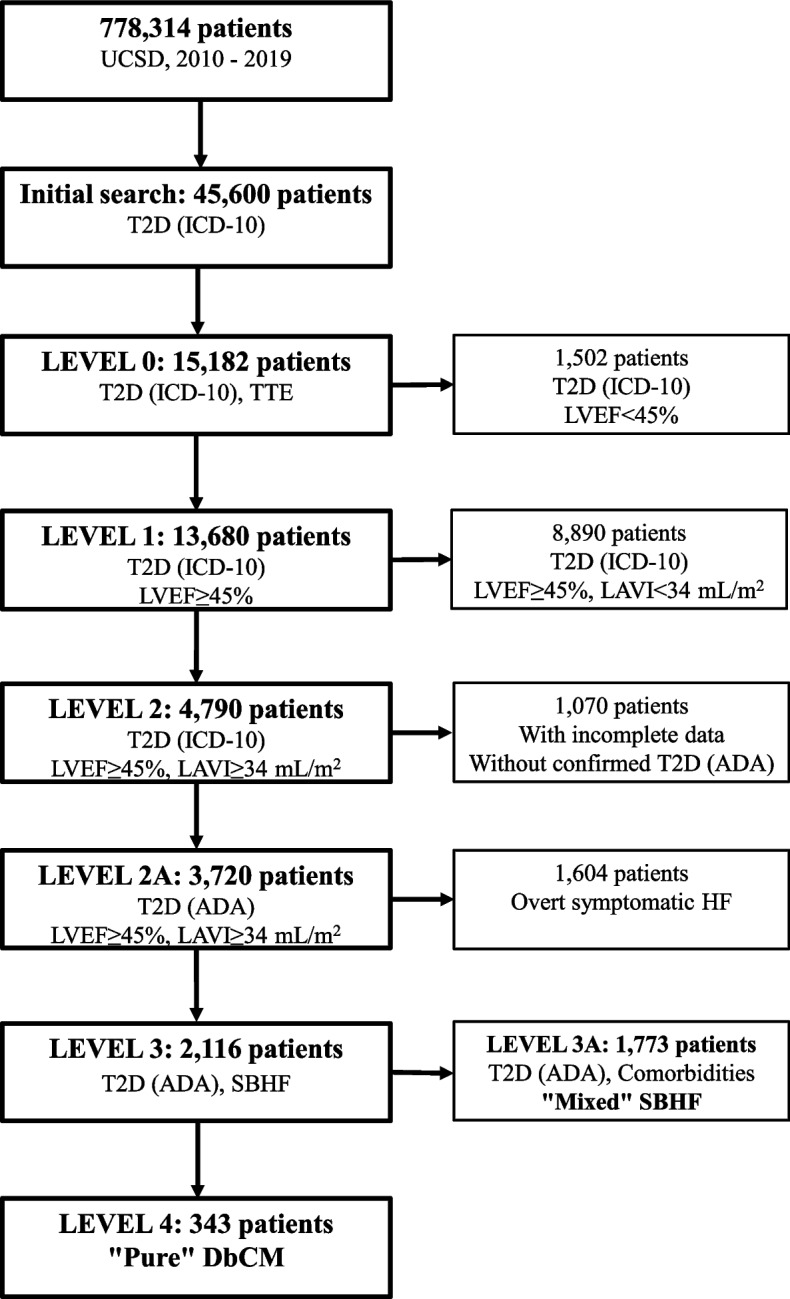
Patient flowchart. Abbreviations: ADA – American Diabetes Association; DbCM – diabetic cardiomyopathy; HF – heart failure; ICD-10 – International Classification of Diseases, Tenth Revision; LAVI – left atrial volume index; LVEF – left ventricular ejection fraction; SBHF – Stage B heart failure; T2D – type 2 diabetes; TTE – transthoracic echocardiogram; UCSD – University of California San Diego Medical Center

Patient flowchart in the study is displayed in Fig. 2.

Of 778,314 patients undergoing medical care at the UCSD in the period from 2010 through 2019, 45,600 patients (5.9%) had diagnosis of T2D according to the ICD-10 (Fig. 2, Initial search). In this group, 15,182 T2D patients (33.3%) had TTE (Fig. 2, Level 0) and, among these patients, 13,680 (90.1%) had LVEF ≥ 45% (Fig. 2, Level 1). Out of them, 4,790 patients had LAVI ≥ 34 mL/m^2^, thus met the echocardiographic criteria of LVD suggesting the diagnosis of DbCM (Fig. 2, Level 2). Based on additional manual review of EMRs, 1,070 patients (22.3% of the entire group of patients with LVD) were excluded because the diagnosis of T2D was not confirmed (643 patients) and/or clinical or echocardiographic data were incomplete (475 patients). Data incompleteness resulted from various causes such as missing results of lab tests, other diagnostic tests, echocardiographic parameters measurements, and BP measurements, as well as insufficient data on the diagnosis and history of comorbidities. The incompleteness of the data was largely due to the medical-record system issues (e.g., some echocardiographic parameters were not available or extractable from EMRs), and not patient-specific characteristics.

With this approach, 3,720 T2D patients with echocardiographic demonstration of LVD suggesting the diagnosis of DbCM were identified (Fig. 2, Level 2A). In this group, 1,604 patients (43.1%) were excluded due to overt symptomatic HF at the time of TTE. Thus, 2,116 asymptomatic T2D patients with SBHF suggesting DbCM were identified (Fig. 2, Level 3). Out of them, 1,773 patients (83.8% of patients with SBHF) were diagnosed with “mixed” SBHF due to coexistence of medical conditions other than T2D which can contribute to SBHF (Fig. 2, Level 3A). Specifically, prevalence of various comorbidities among the T2D patients with “mixed” SBHF was as follows (results are presented according to decreasing values of frequency): severe, moderate, or uncontrolled mild HTN (1,023 patients, 57.7% of patients with “mixed” SBHF), CAD (637 patients, 35.9%), history of acute coronary syndrome (529 patients, 29.8%), coronary revascularization (494 patients, 27.9%) or stroke (431 patients, 24.3%), substance abuse (370 patients, 20.9%), VHD (305 patients, 17.2%), cardiomyopathies of other etiologies (282 patients, 15.9%), history of myocarditis (98 patients, 5.5%) or LVEF < 40% (47 patients, 2.7%), and other medical conditions such as pulmonary arterial hypertension, heart transplant, cardiac involvement in liver cirrhosis or lung and pulmonary vascular diseases and congenital heart disease (182 patients, 10.3%; each condition occurred in < 2% of patients with “mixed” SBHF). Patients with “mixed” SBHF accounted for 47.7% of T2D patients with echocardiographic features of LVD suggesting DbCM. This represents at least 15% of the entire group of T2D patients who underwent echocardiography.

Thus, in the final step of our multi-step approach, we identified 343 patients who met the diagnostic criteria for “pure” DbCM (Fig. 2, Level 4) from the group of 2,116 asymptomatic T2D patients with SBHF suggesting DbCM (Fig. 2, Level 3) and after excluding patients with “mixed” SBHF (Fig. 2, Level 3A). This represents 16.2% of T2D patients with SBHF suggesting DbCM, 9.2% of T2D patients with echocardiographic features of LVD suggesting DbCM who had or did not have symptomatic HF, and at least 2.9% of T2D patients who underwent echocardiography.

Among 3,720 T2D patients with LVD suggesting DbCM who had or did not have symptomatic HF, the following comorbidities that can contribute to LVD were diagnosed (results are presented according to decreasing values of frequency): severe, moderate, or uncontrolled mild HTN (1,696 patients, 45.6% of patients with LVD), CAD (1,560 patients, 41.9%), history of acute coronary syndrome (1,323 patients, 35.6%) or coronary revascularization (1,245 patients, 33.5%), cardiomyopathies of other etiologies (1,044 patients, 28.1%), VHD (885 patients, 23.8%), history of stroke (851 patients, 22.9%), substance abuse (610 patients, 16.4%), LVEF < 40% (488 patients, 13.1%) or myocarditis (207 patients, 5.6%).

In the group of 1,604 T2D patients with LVD suggesting DbCM who had overt HF at the time of TTE (Fig. 2), the following medical conditions that can contribute to LVD were diagnosed (results are presented according to decreasing values of frequency): CAD (923 patients, 57.5% of T2D patients with LVD and symptomatic HF), history of acute coronary syndrome (794 patients, 49.5%) or coronary revascularization (751 patients, 46.8%), cardiomyopathies of other etiologies (762 patients, 47.5%), severe, moderate or mild uncontrolled HTN (673 patients, 42.0%), VHD (580 patients, 36.2%), history of LVEF < 40% (441 patients, 27.5%), stroke (420 patients, 26.2%), substance abuse (240 patients, 15.0%), myocarditis (109 patients, 6.8%) or heart transplant (80 patients, 5.0%). Of 1,604 patients who had LVD and overt HF at the time of TTE, 29 patients (1.8%) had no medical conditions other than T2D that could contribute to LVD.

### Demographic and clinical characteristics of patients with “pure”DbCM

Demographic and clinical characteristics for patients with “pure” DbCM is shown in Table 2.

**Table 2:** Demographic and clinical characteristics of patients with “pure” diabetic cardiomyopathy

Variable	„Pure” DbCM group (*n* = 343)
Age (years)	65.0 (58.5–72.0)
Gender (male/female) *n* (%)	172/171 (50.1/49.9)
Race (White/Asian/African American/Other^a^) *n* (%)	144/58/24/25 (42.0/16.9/7.0/7.3)
Hispanic ethnicity *n* (%)	92 (26.8)
Smoking currently or previously *n* (%)	145 (42.3)
Diagnosis of hyperlipidemia *n* (%)	212 (61.8)
Duration of T2D (months)	45 (11.5–101.5)
Duration of T2D ≥ 10 years *n* (%)	72 (21.0)
Diabetic nephropathy *n* (%)	117 (34.1)
Diabetic retinopathy *n* (%)	57 (16.6)
Nonobstructive coronary artery disease *n* (%)	53 (15.5)
Well controlled mild HTN *n* (%)	238 (69.4)
Chronic kidney disease *n* (%)	128 (37.3)
Peripheral artery disease *n* (%)	18 (5.2)
Nontraumatic lower extremity amputations	13 (3.8)
Atrial fibrillation *n* (%)	88 (25.7)
Stroke of cryptogenic or cardioembolic etiology *n* (%)	13 (3.8)
Body mass index (kg/m^2^)	30.0 (26.0–35.2)
Body mass index ≥ 30 kg/m^2^ *n* (%)	172 (50.1)
Systolic blood pressure (mmHg)	125 (117–132)
Diastolic blood pressure (mmHg)	70 (64–77)
Heart rate (bpm)	75 (65–84)
Hemoglobin (g/dL)	11.8 (10.3–13.5)
Creatinine (mg/dL)	0.94 (0.73–1.29)
Glomerular filtration rate (mL/min/1.73 m^2^)	60 (46–60)
Fasting plasma glucose (mg/dL)	131 (105–169)
Glycated hemoglobin (%)	6.6 (6.1–7.4)
Low-density lipoprotein-cholesterol (mg/dL)	77 (56–101)
High-density lipoprotein-cholesterol (mg/dL)	45 (36–58)
Non-high-density lipoprotein-cholesterol (mg/dL)	106 (83–131)
Triglycerides (mg/dL)	124 (89–174)
Uric acid (mg/dL)	6.2 (5.0–7.5)
ALT (U/L)	19 (14–29)
ASA *n* (%)	174 (50.7)
Metformin *n* (%)	201 (58.6)
Insulin *n* (%)	105 (30.6)
Sulphonylureas *n* (%)	85 (24.8)
DPP-4 inhibitors *n* (%)	39 (11.4)
Thiazolidinediones *n* (%)	21 (6.1)
GLP-1 agonists *n* (%)	8 (2.0)
SGLT-2 inhibitor *n* (%)	1 (0.2)
Beta blocker *n* (%)	156 (45.5)
ACEI *n* (%)	124 (36.2)
ARB *n* (%)	72 (20.9)
Antihypertensive other *n* (%)	144 (42.0)
Diuretic non-potassium-sparing *n* (%)	139 (40.5)
Diuretic potassium-sparing *n* (%)	24 (7.0)
Statin *n* (%)	209 (60.9)
Antilipid other *n* (%)	58 (16.9)
Anticoagulant *n* (%)	64 (18.7)

Data represent the number of patients (*n*) including the percentage of total number (%) or median values with corresponding interquartile range (in parenthesis)

*Abbreviations*: *ACEI* Angiotensin-converting enzyme inhibitor, *ALT* Alanine aminotransferase, *ARB* Angiotensin receptor blocker, *ASA* Acetylsalicylic acid, *DbCM* Diabetic cardiomyopathy, *DPP-4 inhibitors* Dipeptidyl peptidase-4 inhibitors, *GLP-1 agonists* Glucagon-like peptide-1 agonists, *HTN* Essential systemic hypertension, *SGLT-2 inhibitors* Sodium-glucose cotransporter-2 inhibitors, *T2D* Type 2 diabetes

^a^Other races such as Native American, Pacific Islander, and Alaska Native

The population of patients with “pure” DbCM included 50% males and 50% females with an overall median age of 65 years and was racially and ethnically diverse. Comparison of our data with data of the "*2020 Census of Population and Housing*" by the United States Census Bureau on the demographics of San Diego city indicates that racial and ethnic composition of the population of patients with “pure” DbCM in our study is very similar to the population of San Diego city (i.e., White race 42% vs. 41%, Asian race 17% vs. 17%, African American race 7% vs. 6%, Hispanic ethnicity 27% vs. 30%, and Other races, such as Native American, Alaska Native or Pacific Islander, 7% vs. 6%, respectively). Regarding a gender, the population of patients with “pure” DbCM included 50% of females which is also consistent with the female population of San Diego city (49%). The group of patients with “pure” DbCM was characterized by a median duration of T2D of 45 months; however, one fifth of patients have had the diagnosis of T2D for ≥ 10 years. The prevalence of obesity, defined as body mass index ≥ 30 kg/m^2^, was 50%. Cardiovascular risk factors such as hyperlipidemia and smoking (currently or previously) occurred in 42% and 62% of patients, respectively.

Despite patients with severe and moderate HTN were excluded from this analysis, mild HTN was diagnosed in 69% of patients with “pure” DbCM. Most of them, i.e., 232 patients (97.5% of patients with mild HTN) received antihypertensive treatment. Mild HTN was well controlled in this population as evidenced by a median BP of 125/70 mmHg, which meets target BP value recommended for T2D patients [35, 36]. Nonobstructive CAD, peripheral artery disease (PAD), and nontraumatic lower extremity amputations were not common in the studied population (the prevalence of 16%, 5%, and 4%, respectively). While atrial fibrillation occurred in one fourth of the population with “pure” DbCM, prior stroke of cryptogenic or cardioembolic etiology was not common (4% of patients).

The most common diabetic complication in patients with “pure” DbCM was diabetic nephropathy (34% of patients). Of 128 patients (37% of the population with “pure” DbCM) with the diagnosis of CKD, 26 patients (7.6% of the population with “pure” DbCM) were treated with renal replacement therapy and 19 patients (5.5%) received a kidney transplant. Diabetic retinopathy was diagnosed in 17% of patients with “pure” DbCM, including 3 patients (0.8%) with proliferative retinopathy.

While the majority of patients with “pure” DbCM received metformin, one third of them was treated with insulin and one fourth with sulphonylureas (Table 2). Other antidiabetic medications, such as dipeptidyl peptidase-4 (DPP-4) inhibitors, thiazolidinediones, glucagon-like peptide-1 (GLP-1) agonists and sodium-glucose cotransporter-2 (SGLT-2) inhibitors, were administered less frequently. The median level of glycated hemoglobin (HbA1c) of 6.6% indicated a good blood glucose control in the studied population of patients with “pure” DbCM according to the ADA recommendations [35, 36]. A significant portion of patients received guideline-based cardiovascular pharmacotherapies, such as angiotensin-converting enzyme inhibitors (ACEI) and beta blockers. While statins were administered to the majority of patients, the median and mean values of low-density lipoprotein-cholesterol (LDL-C) were 77 (56–101) mg/dL and 81 ± 38 mg/dL, respectively, thus did not meet recommended therapeutic goal for T2D patients [35, 36, 43].

### Echocardiographic characteristics of patients with “pure” DbCM

Echocardiographic characteristics for patients with “pure” DbCM is displayed in Table 3.

**Table 3:** Echocardiographic characteristics of patients with “pure” diabetic cardiomyopathy

Variable	“Pure” DbCM group (*n* = 343)
LVEDd (mm)	47.0 (43.0–50.0)
LAVI (mL/m^2^)	39.0 (35.9–43.7)
LVMI (g/m^2^)	87.7 (73.7–100.7)
LVH *n* (%)	76 (22.2)
LVEF (%)	66 (60–70)
E/A ratio^b^	0.96 (0.8–1.3)
DT (ms)	213 (181–249)
TR velocity^b^ (m/s)	2.6 (2.4–2.9)
TR velocity > 2.8 m/s^b^ *n* (%)	83 (27.8)
E/E’ ratio^a^	11.4 (9.1–14.4)
E/’E’ ratio ≥ 13^a^ *n* (%)	63 (39.4)

Data represent the number of patients (*n*) including the percentage of total number (%) or median values with corresponding interquartile range (in parenthesis)

*Abbreviations*: *A* Peak velocity of transmitral flow during atrial systole, *DT* Deceleration time, *E* Peak velocity of early transmitral flow, *E’* Diastolic mitral annular velocity by Tissue Doppler Imaging, *LAVI* Left atrial volume index, *LVEDd* Left ventricular end-diastolic diameter, *LVEF* Left ventricular ejection fraction, *LVH* Left ventricular hypertrophy, *LVMI* Left ventricular mass index, *TR* tricuspid regurgitation

^a^data was available in 160 patients

^b^data was available in 299 patients

Patients with “pure” DbCM from the studied population were characterized by LAE as evidenced by a median value for LAVI of 39 mL/m^2^, normal LV end-diastolic dimension, and preserved LVEF with a median value for LVEF of 66%. LVH was present in about one fourth of patients with “pure” DbCM. In addition, based on echocardiographic findings, patients with “pure” DbCM were characterized by LVDD of grade II with indeterminate LV filling pressure [33, 34]. However, a significant portion of T2D patients from the studied population had E/E’ ratio ≥ 13 (39% of patients with E/E’ data available) and TR velocity > 2.8 m/s (28% of patients with TR data available), which can indicate elevated LV filling pressure in these patients [33, 34].

## Discussion

This study provides evidence that diabetes-related alterations in cardiac structure and function are highly prevalent in T2D patients in a real-world clinical practice. Specifically, about 16% of T2D patients without overt HF and with echocardiographic features of SBHF had “pure” DbCM that is associated solely with T2D. This represents at least 3% of T2D patients in our study. Moreover, a significant portion of T2D patients with SBHF (about 84%) had “mixed” SBHF that may result from coexistence of T2D and comorbidities such as HTN and CAD which can contribute to SBHF. This represents at least 15% of T2D patients who underwent echocardiography. Notably, LVD defined as LAE in the presence of LVEF ≥ 45% was found in about one third of T2D patients. Most of them (about 57%) had no symptoms of overt HF, thus, could be diagnosed with asymptomatic SBHF. This represents at least 18% of T2D patients.

Our findings emphasize a significance of metabolic-related mechanisms in the pathogenesis of SBHF in patients with T2D. The results of our analysis support a need for early identification of asymptomatic patients with DbCM who can be at increased risk of progression to overt HF and adverse outcome. The unique attribute of our study is the focus on evaluating the occurrence of DbCM in real-world clinical practice. Our study is characterized by consideration of a large population of patients with T2D, application of rigorous methodological approach with well-defined inclusion and exclusion criteria for the study eligibility, identification of a relatively large and demographically diverse population of patients with “pure” DbCM that is associated solely with T2D, quantification of the prevalence of “pure” DbCM as well as “mixed” SBHF, and development of a unique and comprehensive clinical and echocardiographic characteristics of the population with “pure” DbCM. We achieved our goals through reviewing EMRs of a large cohort of T2D patients undergoing contemporary guideline-based medical care provided by the academic medical center. Our findings provide novel insights on the prevalence of DbCM among T2D patients and clinical characteristics of patients with DbCM.

Prevalence of DbCM is difficult to assess due to confounding factors, such as frequently coexisting cardiovascular disease, and a lack of consensus on criteria for defining DbCM [10, 44]. Previous studies on prevalence of DbCM had several limitations, such as heterogenous populations, small sample size, non-uniform set of criteria for DbCM and exclusion criteria identifying factors that may contribute to LVD in T2D patients [8–10, 16, 44]. Consequently, the reported prevalence of DbCM in T2D patients is highly variable (5–58% of patients) [1, 2, 8–10, 16]. Several studies defined DbCM as LVD consisting of both LV systolic dysfunction (LVSD) and LVDD, with or without LVH [8–10, 44]. Existing results indicate that a common form of DbCM is LVDD, often characterized by a restrictive pattern, in the presence of preserved LVEF [4, 8, 9, 16, 45, 46]. In previous studies of asymptomatic T2D populations with various comorbidities, such as HTN (66%-86% of patients) and CAD (19%-36% of patients), 25%-38% of patients had newly detected echocardiographic features of LVDD while only 3% of patients had LVSD [10, 44]. Notably, 7%-28% of patients from these cohorts were diagnosed with HF that was unknown. Multiple echocardiographic parameters and different cut-off values of echocardiographic parameters were used for identifying LVSD and LVDD [8, 9, 16]. A cluster analysis of echocardiographic patterns including various parameters, such as LVMI, E/E’, LVEF and LV volumes, identified three different echocardiographic phenotypes of T2D patients depending on the presence of LV remodeling and subclinical dysfunction [47]. Importantly, these phenotypes were associated with distinct clinical profiles and prognostic significance.

Our approach for diagnosing SBHF related to DbCM was based on identifying LAE in the presence of preserved LVEF [12–15, 18]. Notably, our findings indicate that the vast majority of T2D patients (about 90%) had preserved LVEF, regardless of HF symptoms and comorbidities, which is consistent with the results of previous studies (83–97% of T2D patients depending on the criterion of LVEF that was used) [4, 8–10, 16, 45]. However, in our study, about 30% of T2D patients with preserved LVEF had LAE as evidenced by elevated LAVI (defined as LAVI ≥ 34 mL/m^2^). An increase in LA size and volume is among key structural alterations characterizing structural heart disease which can support the diagnosis of SBHF in the presence of preserved LVEF [15]. LAE was previously shown to be a common echocardiographic abnormality in patients with preserved LVEF including T2D populations [7, 16, 17, 21, 23–26, 48]. For example, the prevalence of LAE in our study is similar to the 35% rate of LAE (defined as LAVI ≥ 32 mL/m^2^) in the study of Wang et al. [16] including asymptomatic T2D patients with preserved LVEF. Based on our findings, at least 40% of T2D patients, regardless of HF symptoms and comorbidities, had echocardiographic features of LVD such as LVSD defined as LVEF < 45% or LAE in the presence of preserved LVEF (about 10% and 30% of patients, respectively).

Our findings also indicate that asymptomatic T2D patients with “pure” DbCM and preserved LVEF (median LVEF of 66%) had at least moderate LVDD. However, about one third of this population had echocardiographic features of more severe LVDD as evidenced by E/E’ ≥ 13. This represents higher incidence of elevated E/E’ compared to the 10% rate in the study of Wang et al. [16] despite an older age of patients in that study. Also, E/E’, LAVI, and E/A values were higher in patients with “pure” DbCM in our study compared to other studies [8, 16]. These abnormalities may indicate the presence of elevated LV filling pressure and more severe LVDD in patients with “pure” DbCM in our study compared to other studies. The LVEF value and LVH rate in our study were comparable to those reported by other studies on DbCM or SBHF in T2D patients (e.g., [16]).

With our approach based on a well-defined set of inclusion and exclusion criteria, we identified the group of asymptomatic 343 T2D patients with “pure” DbCM that was associated solely with T2D. This represents the larger DbCM cohort compared to previous studies, for example 23 patients with DbCM [8], 33 patients with DbCM [9], and 169 patients with DbCM and HTN [16]. The differences between the studies [8, 9, 16] and our study, especially in terms of methodological details, are important as they could affect the study findings. The use of various criteria for defining LVD and no exclusion of patients with comorbidities could impact the DbCM rates reported in several studies [4, 8–10, 44, 45].

In the study of Dandamudi et al. [8] of 2042 randomly selected individuals, 17% met the criteria for DbCM and 54% had LVDD from the group of T2D patients without overt HF and a history of cardiovascular disease. Patients with DbCM accounted for 1.1% of the community population. Compared to our study, the study [8] had smaller sample size (136 T2D patients and 23 DbCM patients including 26% of females), used different methodology for identification of DbCM (defined as LVSD with LVEF < 50% or at least moderate LVDD), and assessed comorbidities such as CAD and HTN using community medical records. These methodological differences can contribute to higher prevalence of LVDD and DbCM in [8] compared to our study. In addition, the DbCM patients in [8] were older, had lower LVEF, and higher LVMI and creatinine level, and were subjected to medical care in an earlier period of 1997–2000.

In the study of Pham et al. [9] of 656 asymptomatic T2D patients, DbCM defined as the presence of LVH, LV dilation, LVSD or LV wall motion abnormalities in the absence of HTN and CAD, was diagnosed in 5% of patients. It is conceivable that this relatively low prevalence of DbCM resulted from a methodological approach lacking the LVDD criteria for identification of DbCM and a relatively small sample size. Notably, this prevalence of DbCM was reported although the population had a long-standing T2D (mean duration of 14 years), poor T2D control (mean HbA1c of 8.7%), mandatory additional cardiovascular risk factor (such as dyslipidemia, HTN, microalbuminuria, PAD), and was treated within the period of 1991–2008.

In the study of Wang et al. [16], 58% of 290 asymptomatic T2D patients had SBHF defined as LVDD based on E/E’ > 13, LAE based on LAVI ≥ 32 ml/m^2^, global longitudinal strain < 16%, or LVH. This studied population had well controlled T2D (mean HbA1c of 5.6%), LVEF ≥ 50%, and no evidence of CAD, HF, history of LVEF < 40%, and VHD. However, the HTN was not used as an exclusion criterion and 77% of patients were diagnosed with HTN with the mean BP of 139/81 mmHg (vs. 125/70 mmHg in our study). This factor along with relatively old patients’ age (mean of 71 years), heart disorders in the past medical history in 15% of patients, and a wide range of echocardiographic criteria for SBHF could contribute to relatively high prevalence of SBHF and it is difficult to interpret the results in [16] in terms of the prevalence of DbCM.

Our findings indicate that “mixed” SBHF is common in T2D patients without overt HF and was identified in at least 15% of T2D population with a median age of 65 years. Severe, moderate, or uncontrolled mild HTN (58% of patients) and significant CAD (36%) were among the most common comorbidities in T2D patients with “mixed” SBHF. While quantification of contributions of specific comorbidities to SBHF in T2D patients has not been well established, identification of patients with “mixed” SBHF and potential application of treatments targeting diabetic-related cardiac abnormalities may prevent further cardiac damage in this specific T2D population.

DbCM is associated with an increased risk of progression to overt HF. It was previously shown that up to 24% of patients with DbCM/SBHF progress to SCHF or death within 1.5 years and 37% within 5 years [2, 45]. In the study of Dandamudi et al. [8], 31% of patients with DbCM died or developed HF at 9 years. Based on our findings, about 43% of T2D patients with LVD and comorbidities had symptomatic HF at the time of TTE, which represents at least 14% of T2D patients who underwent echocardiography. An incidence of overt HF in the entire T2D population was previously shown to be as high as 22% and correlated with older age, CAD, poor glycemic control, and high BMI, which were found to be predictors of HF development [28–30].

We determined that in real-world clinical practice only about 33% of T2D patients underwent echocardiography. Our findings indicate that routine echocardiography is highly desirable for early diagnosis of DbCM and may ultimately improve clinical outcome. Regular echocardiographic assessment should be recommended especially in asymptomatic T2D patients characterized by specific clinical features indicating an increased risk of developing “pure” DbCM such as age > 65 years, duration of T2D ≥ 4 years, obesity, elevated BP, CKD, microvascular complications such as diabetic nephropathy, and non-optimal LDL-C (Table 2). Moreover, in our study, “pure” DbCM was identified in patients with a relatively short duration of T2D compared to other studies [8, 9, 16]. It is notable that even T2D patients treated according to evidence-based guidelines resulting in a good control of HbA1c, BP, and LDL-C were diagnosed with SBHF (Table 2). Also, based on our findings, elevated LAVI, E/E’( ≥ 13), and TR velocity may support the diagnosis of DbCM, especially in T2D patients with preserved LVEF. Overall, in our study, 10% of patients with initial diagnosis of SBHF ultimately satisfied the diagnostic criteria for “pure” DbCM.

Therapies that target metabolic derangement responsible for DbCM can be useful in T2D patients with SBHF to prevent progression to symptomatic HF, particularly in patients with “pure” DbCM associated solely with T2D [12, 13, 18]. These therapies may be also useful in patients with “mixed” SBHF. There is a clear unmet need to develop effective therapies that target diabetes-related cardiac disorders for preventing DbCM and developing SCHF [3, 12, 13, 18, 49, 50].

The strengths of our study are associated with the approach related to search strategy, selection criteria, and data quality control. Important aspects related to the methodology of our study include the large sample size of T2D patients who underwent TTE, well-defined inclusion and exclusion criteria, and availability of various clinical and echocardiographic data for identifying and characterizing a relatively large population of patients with “pure” DbCM. In addition, this population was racially and ethnically diverse and included an equal percentage (50%) of female and male patients. This research was conducted in the academic medical center that ensures high quality medical care based on guideline-based therapies. Importantly, automated EMRs search and diagnosis of T2D, HF, and comorbidities were validated through manual search and thorough review of EMRs. However, because of retrospective nature of the study, the review of data has been limited to T2D patients who had obtained TTE in the past for various reasons. Implementation of two echocardiographic criteria (LVEF ≥ 45%, LAVI ≥ 34 mL/m^2^) for selection of DbCM patients is believed to be weakly limiting because of major significance and frequent prevalence of these features in T2D patients with SBHF [12, 13, 15, 16]. However, the percentages related to the prevalence of DbCM may be considered as conservative estimates. The use of multiple echocardiographic criteria compared to the use of a single criterion of LAE could provide advantage for identification of DbCM [17, 51]. Also, underlying mechanisms of the development of DbCM have not been well established and require further basic and clinical studies [52–55].

## Conclusions

Our findings provide insights into prevalence of DbCM in the population of patients with T2D in real-world clinical practice. We indicate that DbCM affects a significant portion of T2D patients. We determined that about 16% of T2D patients with asymptomatic SBHF had “pure” DbCM that is associated solely with T2D. This represents at least 3% of the entire T2D population in our study. Most T2D patients with SBHF (about 84%) had “mixed” SBHF that may result from coexistence of T2D and other comorbidities, such as HTN and CAD. This represents at least 15% of the entire T2D population.

Our results have important implications for clinical practice. Identification of patients with DbCM among T2D patients can be critical for improving their clinical outcome. Our findings indicate that routine echocardiography is underutilized in the management of T2D patients; however, it is highly desirable for the diagnosis of DbCM. Application of single echocardiographic criterion of elevated LAVI may support the diagnosis of SBHF in asymptomatic T2D patients with preserved LVEF. However, future studies in which multiple echocardiographic parameters are available for identifying DbCM are needed. In addition, further prospective long-term clinical studies of the diagnostic criteria and prevalence of DbCM, clinical characteristics of DbCM population, and prognostic factors of development of DbCM and overt HF in patients with T2D are required.

## Data Availability

The data presented in this study are available on request from the corresponding author. The data are not publicly available due to privacy restrictions.

## Abbreviations

BP: Blood pressure
CAD: Coronary artery disease
CKD: Chronic kidney disease
DbCM: Diabetic cardiomyopathy
E/E’: Peak velocity of early transmitral flow/diastolic mitral annular velocity by TDI
EMRs: Epic electronic medical records
HbA1c: Glycated hemoglobin
HF: Heart failure
HTN: Essential systemic hypertension
LA: Left atrium
LAE: Left atrial enlargement
LAVI: Left atrial volume index
LDL-C: Low-density lipoprotein-cholesterol
LV: Left ventricle
LVD: Left ventricular dysfunction
LVDD: Left ventricular diastolic dysfunction
LVEF: Left ventricular ejection fraction
LVH: Left ventricular hypertrophy
LVMI: Left ventricular mass index
LVSD: Left ventricular systolic dysfunction
PAD: Peripheral artery disease
SBHF: Stage B heart failure
SCHF, SDHF: Stage C (D) heart failure
T2D: Type 2 diabetes
TDI: Tissue Doppler Imaging
TR: Tricuspid regurgitation
TTE: Transthoracic echocardiogram
UCSD: University of California San Diego Medical Center
VHD: Valvular heart disease
